# The MITRE trial protocol: a study to evaluate the microbiome as a biomarker of efficacy and toxicity in cancer patients receiving immune checkpoint inhibitor therapy

**DOI:** 10.1186/s12885-021-09156-x

**Published:** 2022-01-24

**Authors:** Nicola A. Thompson, Grant D. Stewart, Sarah J. Welsh, Gary J. Doherty, Matthew J. Robinson, B. Anne Neville, Kevin Vervier, Simon R. Harris, David J. Adams, Katy Dalchau, David Bruce, Nikolaos Demiris, Trevor D. Lawley, Pippa G. Corrie

**Affiliations:** 1 Department of Oncology, Cambridge University Hospitals NHS Foundation Trust, Cambridge, UK; 2 Department of Surgery, University of Cambridge, Cambridge, UK; 3 Microbiotica, Chesterford Research Park, Cambridge, UK; 4 Wellcome Sanger Institute, Cambridge, UK; 5 Cambridge Clinical Trials Unit – Cancer Theme, Cambridge University Hospitals NHS Foundation Trust, Cambridge, UK

**Keywords:** Microbiome, Immunotherapy, Melanoma, Renal cancer, Non-small cell lung cancer, Biomarker, Immune checkpoint inhibitor, Efficacy, Toxicity

## Abstract

**Background:**

The gut microbiome is implicated as a marker of response to  immune checkpoint inhibitors (ICI) based on preclinical mouse models and preliminary observations in limited patient series. Furthermore, early studies suggest faecal microbial transfer may have therapeutic potential, converting ICI non-responders into responders. So far, identification of specific responsible bacterial taxa has been inconsistent, which limits future application. The MITRE study will explore and validate a microbiome signature in a larger scale prospective study across several different cancer types.

**Methods:**

Melanoma, renal cancer and non-small cell lung cancer patients who are planned to receive standard immune checkpoint inhibitors are being recruited to the MITRE study. Longitudinal stool samples are collected prior to treatment, then at 6 weeks, 3, 6 and 12 months during treatment, or at disease progression/recurrence (whichever is sooner), as well as after a severe (≥grade 3 CTCAE v5.0) immune-related adverse event. Additionally, whole blood, plasma, buffy coat, RNA and peripheral blood mononuclear cells (PBMCs) is collected at similar time points and will be used for exploratory analyses. Archival tumour tissue, tumour biopsies at progression/relapse, as well as any biopsies from body organs collected after a severe toxicity are collected. The primary outcome measure is the ability of the microbiome signature to predict 1 year progression-free survival (PFS) in patients with advanced disease. Secondary outcomes include microbiome correlations with toxicity and other efficacy end-points. Biosamples will be used to explore immunological and genomic correlates. A sub-study will evaluate both COVID-19 antigen and antibody associations with the microbiome.

**Discussion:**

There is an urgent need to identify biomarkers that are predictive of treatment response, resistance and toxicity to immunotherapy. The data generated from this study will both help inform patient selection for these drugs and provide information that may allow therapeutic manipulation of the microbiome to improve future patient outcomes.

**Trial registration:**

NCT04107168, ClinicalTrials.gov, registered 09/27/2019.

Protocol V3.2 (16/04/2021).

**Supplementary Information:**

The online version contains supplementary material available at 10.1186/s12885-021-09156-x.

## Background

### The gut microbiome during health and cancer

A healthy individual harbours some 300–500 bacterial species in their gastrointestinal microbiome [1]. While some bacterial genera are common between individuals, the complete composition at strain level taxonomy within any one person is unique, often compared to a “fingerprint”. Our understanding of the human intestinal microbiome, primarily using sequence-based approaches, has developed greatly in recent years. Bacteria make up the majority of the microbial biomass within stool samples and play a central role the development and regulation of our mucosal and systemic immune systems, digestion of food and sustenance, and resistance to pathogens [2]. Pathological imbalances in the microbial community, termed dysbiosis, can be caused by diet, drugs, genetics and infection. Intestinal dysbiosis is linked to a growing list of infectious diseases, autoimmune diseases and syndromes, and could potentially impact the clinical response to therapies, particularly cancer immunotherapy.

The link between human-associated bacteria, cancer development and immunotherapy is not new and pre-dates our recent realization that the human microbiome plays a pervasive role in our health and disease. For example, specific bacterial species are known to cause certain types of cancers, such as gastric cancer caused by *Helicobacter pylori*, while some bacterial agents such as Coley’s Toxin (*Streptococcus pyogenes*) and BCG vaccination have been shown to alter the host response to cancer [3–5]. The mechanisms of action for Coley’s toxin and BCG vaccination remain largely unknown, but these observations highlight an opportunity to use bacteria therapeutically to prevent or treat cancer. Recent evidence suggest that a patient’s intestinal microbiota composition plays a critical, though as yet poorly defined, role in determining both therapeutic efficacy and likelihood of significant adverse events to T-cell checkpoint inhibitor immunotherapy [6–11].

### The gut microbiota as a predictive biomarker for immune checkpoint inhibitor response and toxicity

Immune checkpoint inhibitors (ICIs) are revolutionising treatment of many types of metastatic cancer and signals of efficacy in earlier stages of disease are now emerging. Anti-PD-(L)1 ± anti-CTLA-4 antibodies are now routinely used to treat patients with metastatic cancers, including melanoma [12, 13], renal cancer [14, 15], and non-small cell lung cancer (NSCLC) [16, 17] in expectation of improving overall survival. ICIs are being tested in multiple other cancer types and many new licenses for use are anticipated in the coming years. The first adjuvant ICI trials in patients with resected melanoma at high risk of recurrence have reported improvements in relapse-free survival [18, 19] and adjuvant checkpoint blockade is now being tested in other cancers. While these early indications offer great hope for improving outcomes for cancer patients, ICIs are not without their limitations. Firstly, not all patients respond: response rates vary between 25 and 60% at best, depending on treatment regimen and cancer type; thus most patients embarking on treatment will not benefit [20]. Secondly, immune-related toxicities occur, which are complex, unpredictable, and may be severe or life threatening in up to 50% of treated patients [20]. Thus, significant numbers of patients will be hospitalised to manage toxicity, or require long term (sometimes lifelong) supportive therapies for permanent damage to body systems.

A number of studies using one or two cohorts have associated the gut microbiome with response to ICIs in advanced melanoma, NSCLC and renal cancer [21]. Furthermore, two interventional studies in advanced melanoma have demonstrated that faecal microbiota transplant (FMT) can convert patients that have previously progressed on anti-PD1 immunotherapy to become responsive [22, 23]. Interestingly, ICI-triggered toxicities are also linked to the patients’ microbiome [21] and there is also potential for FMT to ameliorate toxicities [24]. While the mechanisms of efficacy and toxicity remain to be defined, positive clinical outcomes from ICIs may involve stimulation of intestinal dendritic cells and circulating T cells by specific taxonomic groups of intestinal bacteria [8, 25]. Understanding the role of the microbiome may be valuable to guide patient therapy in the future [26] and to develop co-therapies to increase the efficacy of ICIs [21].

Most human microbiome studies performed in cancer patients on immunotherapy have been statistically underpowered and have mainly relied on 16S rRNA gene sequencing, which has taxonomic resolution limited to genus level. Since many beneficial traits in symbiotic bacteria can be distinct to strain level, these approaches most likely provide neither the strain level taxonomic and functional resolution, nor the biological information needed to accurately identify bacterial biomarkers to predict beneficial or detrimental bacterial strains. We have used temporal, deep shotgun metagenomic profiling in a pilot cohort of metastatic melanoma patients treated with ICIs. This was performed using Microbiotica’s platform that comprises the leading Reference Genome Database to give the most comprehensive and precise profiling of the gut microbiome. This gives the resolution necessary to identify bacteria species and even strains linked with outcome. Further, we have been able to show representative strains of the species associated with response have anti-tumour efficacy in vitro and in vivo [27].

We aim to expand knowledge beyond prior studies, both because of the high resolution achieved and because our workflow allows us to culture and archive individual bacterial strains at scale, thus allowing us to test hypotheses using the organisms isolated and identified. Our approach uses large-scale metagenomics of patient cohorts and sample banking, progressing to hypothesis testing using functional studies to fulfil Koch’s postulates for both beneficial and pathogenic bacteria. We will access a variety of in vitro and in vivo pre-clinical models to validate and test beneficial and pathogenic bacteria on host responses, specifically in relation to immunity and tumour growth as well as drug efficacy and toxicity. These functional platforms are critical to develop and progress translational opportunities to enable future patient selection based on microbiome biomarkers to personalise cancer immunotherapy, as well as to precisely manipulate a patient’s microbiota to optimise cancer immunotherapy. Comparison with a limited cohort of healthy household members acting as controls will provide additional essential information about the role of the patient-specific microbiome.

### Impact of COVID-19

COVID-19, also termed severe acute respiratory syndrome coronavirus 2 (SARS-CoV-2), is caused by a coronavirus that is phylogenetically similar to SARS-CoV, the agent of Severe Acute Respiratory Syndrome. Coronaviruses are enveloped positive-sense RNA viruses. In humans, COVID-19 infection is primarily a respiratory disease which can vary from asymptomatic infection through coryzal upper respiratory infection to severe respiratory distress and death. Studies of biomarkers predicting for outcomes from COVID-19 infection have identified several factors associated with immunity including lymphocyte count, neutrophil count, and inflammatory markers such as C-reactive protein, Interleukin-6 and Procalcitonin [28]. In addition, worse outcomes are seen with older age and in males, smokers and diabetics [29]. It is reasonable to assume that COVID-19 may influence (and potentially be influenced by) the microbiome, and therefore we will assess patient COVID-19 status as part of this study.

National arrangements for population and cancer patient COVID-19 testing are evolving and are likely to continue to change during the course of the study. We wish to evaluate both COVID-19 antigen and antibody associations with the microbiome. We will aim to use results available from routine testing where possible, but in the absence of routine testing for either or both of these entities, we will include the option to test within this protocol.

### Rationale for the MITRE study

The pre-treatment presence (and prevalence), or absence of, specific bacterial strains may dictate response and toxicity to immune checkpoint inhibitors.

This hypothesis will be explored and validated in a large-scale prospective study of biological samples collected from patients receiving immune checkpoint inhibitors, as well as household control participants, using our metagenomics platform with matched clinical data.

## Methods/design

### Study design

**Fig. 1:**
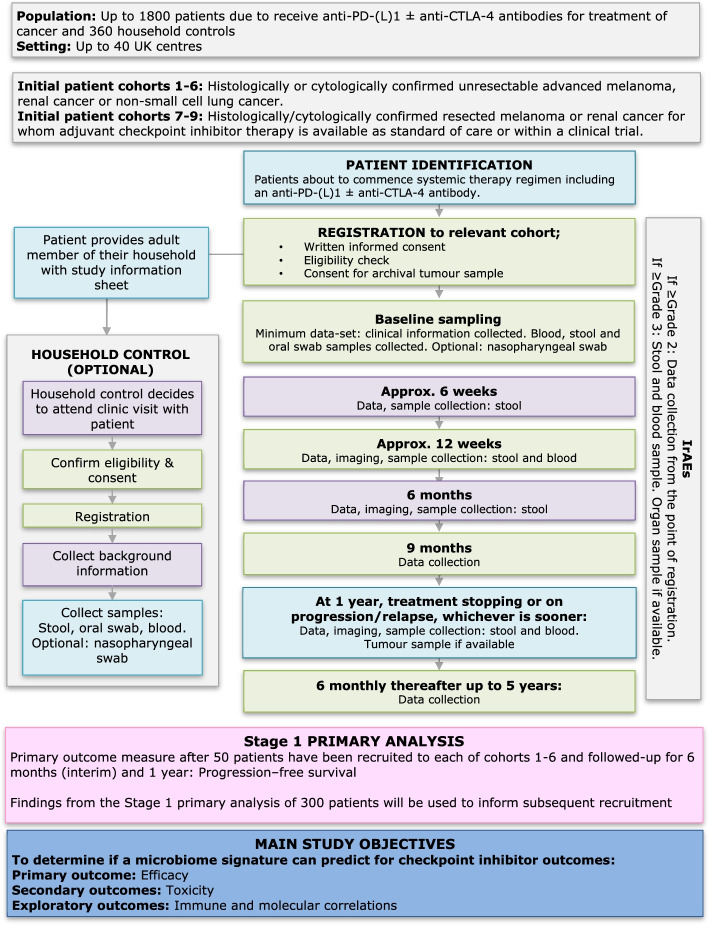
MITRE Study Flow Chart

This is a multi-centre, non-interventional study, involving up to 40 UK sites. Up to 1800 cancer patients and 360 household control participants will be enrolled (Fig. 1).

Cancer patients due to commence standard treatment with systemic therapy including an anti- PD- (L)1 ± anti-CTLA-4 antibody will be invited to take part in this multi-cohort, multi-centre study.

Consenting patients will be asked to provide clinical information and donate biological samples before, during and after completing their treatment (see section Additional file 1), for the duration of the study period, up to a maximum of 5 years.

Consenting patients will also be asked to invite a member of their household to provide limited demographic, health and lifestyle information and biological samples at a single time point only (see Additional file 2).

### Study objectives

#### Primary objective

- The primary objective is to assess whether there is a gut microbiome ‘signature’ which can predict for ICI treatment efficacy in patients with advanced, unresectable cancers.

#### Secondary objectives

- To determine if the gut microbiome signature predicts for ICI risk of relapse in patients with resected cancers.
- To evaluate any association with the gut microbiota and ICI-induced toxicity.
- To assess whether certain medicines (including antibiotics, steroids, proton pump inhibitors (PPIs), non-steroidal analgesics (NSAIDs), probiotics taken within the past 6 months and/or during treatment) affect the gut microbiome signature.
- To assess whether patients’ diet and body mass index (BMI) affects the gut microbiome signature.
- To compare and contrast the oral and gut microbiome of patients and their household controls.
- To study the effects of ICIs on the gut microbiota over time.
- To establish a unique microbiome biobank of longitudinal samples available for future interrogation.

#### Exploratory objectives

- To correlate microbiome findings with patients’ peripheral blood immune and cytokine profiles (including IFNγ, TNFα, IL-6) measured in plasma and PBMCs collected prior to and during treatment.
- To assess pre-treatment and on-treatment/post-treatment tumour samples for the immunoscore and other immune signatures, including expression of proteins potentially linked to checkpoint inhibitor response, toxicity and immunity.
- To measure genomic/transcriptomic alterations in blood (including germ-line) and tumours, including (but not limited to) assessment of tumour mutational burden, single nucleotide variants (SNVs) in immune-related genes and HLA status.
- To measure the presence and nature of any circulating bacterial nucleotides in study patients.
- To generate primary cell lines from patient PBMCs and tumour tissue to undertake functional immune assays.
- To explore any interaction between COVID-19 status and the microbiome

### Study outcome measures

#### Primary outcome measure

**Fig. 2:**
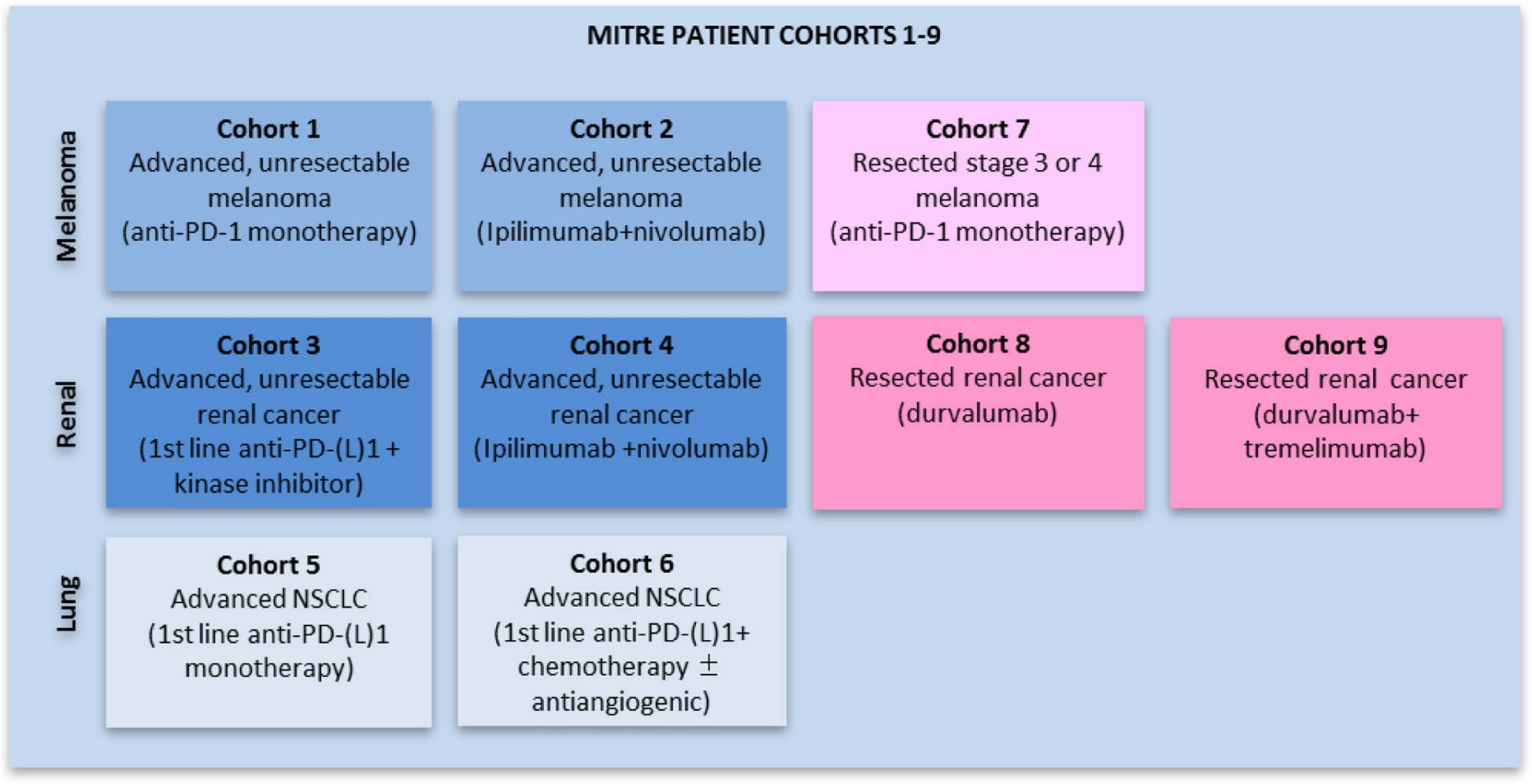
MITRE Patient Cohorts 1–9

Based on pilot data and using an iterative process, a putative microbiome signature was defined [27] and will be prospectively evaluated for its ability to stratify cancer patients receiving ICIs into treatment responders and non-responders. The primary outcome measure is the ability to predict for progression-free survival (PFS) of > 1 year. The primary outcome will be determined for an initial series of patient cohorts limited to advanced melanoma, renal cancer and non-small cell lung cancer (NSCLC) (Table 1 and Fig. 2).

**Table 1:** Cohorts 1–6 – Advanced disease cohorts and defined standard of care ICI-containing treatment regimens

Cohort	Disease	Regimens	Primary outcome measure
Cohort 1	Unresectable AJCC stage 3 or 4 melanoma	Anti-PD-1 monotherapy (nivolumab or pembrolizumab)	1 year PFS
Cohort 2	Unresectable AJCC stage 3 or 4 melanoma	Ipilimumab+nivolumab	1 year PFS
Cohort 3	Advanced renal cell carcinoma	Anti-PD-(L)1 + kinase inhibitor	1 year PFS
Cohort 4	Advanced renal cell carcinoma	Ipilimumab+nivolumab	1 year PFS
Cohort 5	Advanced NSCLC	Anti-PD-(L)1 monotherapy in the first-line setting	1 year PFS
Cohort 6	Advanced NSCLC	Anti-PD-(L)1 + chemotherapy ± antiangiogenic in the first-line setting	1 year PFS

#### Secondary outcome measures

- The ability of the microbiome signature to predict 6 month PFS, 2 year PFS, overall response rate, median PFS and median overall survival (OS) in cohorts 1–6.
- The ability of the microbiome signature to predict for 1 and 2 year relapse after resection of high risk melanoma or renal cancer in cohorts 7–9. (Table 2 and Fig. 2)

**Table 2:** Cohorts 7–9 – Adjuvant disease cohorts and defined standard of care ICI-containing treatment regimens

Cohort	Disease	Regimen	Primary outcome measure
Cohort 7	Resected AJCC stage 3 or 4 melanoma	Anti-PD-1 monotherapy (nivolumab or pembrolizumab)	1 year & 2 year RFS
Cohort 8	Resected renal cancer	Durvalumab	1 year & 2 year RFS
Cohort 9	Resected renal cancer	Durvalumab+tremelimumab	1 year & 2 year RFS

- To compare oral and gut microbiome findings and their association with treatment efficacy.
- To correlate microbiome findings with incidence and characteristics of CTCAE V5-defined Grade > 3 IrAEs in all enrolled patients, and any association with response to immunosuppressants.
- To correlate microbiome findings with aspects of pre-existing patient characteristics and behaviour including but not limited to diet, smoking history, BMI, use of antibiotics, steroids, PPIs, NSAIDs and probiotics.
- To compare the microbiome signature of cancer patients with a household control group of people who are not known to have cancer.
- To retain a library of biological samples with linked patient data for future research.

#### Exploratory outcome measures

- To correlate the gut microbiome findings with the patient’s immune status (HLA subtype, auto-antibodies)
- To correlate the gut microbiome findings with peripheral immune cell subset composition, surface antigen expression and peripheral blood cytokine (including IFNγ, TNFα, IL-6) and chemokine expression
- To correlate the gut microbiome findings with pre-treatment – and where possible, on-treatment - intratumoural immune cell infiltrate (immunoscore) and microenvironment (including but not limited to CD4, CD8, FOXP3, PD-1, PD-L1, Granzyme B expression)
- To correlate the gut microbiota findings with tumour mutational burden (measured in tissue and/or circulating free DNA).
- To undertake next generation sequencing of a defined immune gene panel and assess interactions with the microbiome signature.
- To measure the presence and nature of any circulating bacterial nucleotides in study patients.
- To correlate the gut microbiome findings with expression of immune signatures measured using RNAseq, T cell receptor and B cell receptor clonality
- To generate primary cell lines from patient PBMCs and tumour for functional immune assays
- To correlate microbiome findings with COVID-19 status (antigen and/or antibodies detected in nasopharyngeal swab, blood and/or stool)

### Recruitment of patients and eligibility

#### Study population

MITRE is recruiting adult (≥18 years) patients due to commence either adjuvant or palliative treatment with systemic therapy including an anti-PD-(L)1 antibody ± anti-CTLA-4 antibody with a diagnosis of either melanoma, renal cancer, or non-small cell lung cancer. Patients are recruited to 9 cohorts, according to tumour type, treatment and disease stage (Fig. 2).

Patients with advanced disease must have measurable lesions identified within 45 days prior to starting treatment by formal cross-sectional imaging, or clinical measurements. For patients with resected disease, full body imaging must be performed within 12 weeks of planned treatment start date showing no active disease.

Patients with advanced disease must have received no prior ICI for advanced disease. Previous treatment with other types of anti-cancer therapy is allowed, with the exception of NSCLC patients being recruited to cohorts 5 and 6. Prior (neo) adjuvant therapy with ICIs is allowed.

Patients are excluded if they have one or more additional different invasive malignancy diagnosed within the last year not in complete remission, or an additional significant medical or psychiatric condition which would place the patient at undue risk (such as uncontrolled ischaemic heart disease, inflammatory bowel disease, pregnancy/lactation). Regular requirement for non-physiological doses of oral steroids, or regular use of any other immunosuppressive agents is not allowed; regular requirement for prednisolone at a dose of 10 mg or less, or equivalent doses, are allowed. Use of inhaled or topical steroids is also allowed.

All patients must provide written informed consent at registration.

#### Household controls

Cancer patients who consent to take part in the MITRE study will be asked to invite an adult member of their household (age ≥ 18 years) to also take part in this study, to act as a limited control group (up to 360 people will be recruited to this group).

These controls must not have had any gastrointestinal infections in the last 6 months, or have any inflammatory intestinal disease. They must not have taken any antibiotics in the past 6 months, have a history of active cancer, chronic autoimmune disease, significant allergies or an episode of COVID-19 infection requiring hospital admission. Additionally, they should not be taking regular steroids, PPIs, or NSAIDs.

#### Recruitment process

Eligible patients will be invited to participate in this study. Consenting patients will be registered centrally at the Cambridge Clinical Trials Unit – Cancer Theme (CCTU-CT). Patients will be identified in the specialist clinics at the participating centres as per local practice.

### Patient registration

Eligibility criteria must be met before registering the patient on the study. Consenting patients will be registered centrally at the Cambridge Clinical Trials Unit – Cancer Theme (CCTU-CT).

Upon registration, the investigator or designee, must complete the registration electronic CRF (eCRF). Upon completion and submission of these eCRFs the CCTU-CT trial coordinator will check registration eCRFs and assign a unique study ID. This study ID should be used in all future correspondence and patient-related documents.

Any source data transferred should be anonymised to unique study ID, date of birth and patient initials.

### Assessments on study

Patients will be followed up during their routine clinic visits, with study-specific data collection at time points approximating to baseline (pre-treatment), 6–8 weeks, 12 weeks, 3 monthly until 1 year, then 6 monthly thereafter until and including disease progression, or relapse, or study termination.

Immune-related adverse events (IrAEs) will be recorded at each clinic visit.

#### Imaging assessments

Patients will undergo radiological assessment for measurable disease during treatment as per local practice, but is recommended to be done approximately every 12 weeks while on treatment for patients with advanced disease and approximately every 6 months for those patients receiving adjuvant therapy. Imaging frequency after stopping treatment is as per local practice.

The following scans will be reported using RECIST 1.1 criteria for patients with advanced disease:

- Baseline, pre-treatment scan
- For patients on treatment, or off treatment but progression-free: approximately 12 weeks, 6 months, 1 year
- At the time of disease progression, if different to these time-points

Once progression has been confirmed by RECIST 1.1 criteria, further RECIST measurements are not required for the purpose of this study.

### ICI treatment

Specific details of the systemic therapy regimens being used to treat patients taking part in this study are not mandated in this protocol, since this is an observational study. However, all drugs (anti-cancer drugs) as well as any radiotherapy and surgery used to treat cancer during the study period will be recorded in the CRF.

Patients experiencing adverse events while on treatment are managed according to local guidelines. No routine adverse event reporting is being undertaken as part of this protocol.

Documentation of a possible/probable/definite IrAE of CTCAE grade > 3 will trigger the requirement to collect a stool and blood sample within 2 weeks of documentation (Additional file 1).

### Additional treatment

Patients can receive full supportive care during and after the administration of ICIs. Palliative radiotherapy, surgery, corticosteroids/other immunosuppressive agents, transfusion with blood products and bisphosphonates are all allowed.

### Treatment for disease progression or relapse

Treatment for disease progression in the metastatic setting and for relapse on or after adjuvant therapy is at the Investigator’s discretion and local practice. Patients deriving clinical benefit from palliative therapy with ICIs in the presence of progression determined by RECIST 1.1 may continue on treatment beyond progression at the investigator’s discretion. However, no further formal RECIST reporting will be required.

For patients with advanced disease, the first RECIST progression date will be used for measuring PFS in this study. For adjuvant patients, the first date of relapse will be used for measuring RFS.

### Collection of clinical data

No additional clinic visits or consultations are required for this study over and above standard of care. Cancer patients will be followed up during their routine clinic visits/consultations, with study data collection time points approximating to baseline (pre-treatment), 6–8 weeks, 12 weeks, 3 monthly up to 1 year, then 6 monthly thereafter until disease progression or relapse, up until the study closure date. Additional clinic assessments may be required as per local practice but study data collection will only be required if the assessment is for first disease progression/relapse. Details of data collected at each visit is available in Additional file 3.

### Biological sample collection

A stool sample, oral swab sample, blood samples and routine laboratory data (as per local practice) will be collected at baseline (pre-treatment).

Further stool samples will be collected at approximately 3–6 weeks (ahead of the 6–8 week visit), 9–12 weeks (ahead of the 12 week visit), approx. 6 months, at the time of a CTCAE grade > 3 IrAE, at disease progression, relapse or at 1 year, whichever occurs soonest.

Further blood samples will be taken at a clinic visit approximating to 12 weeks, at 1 year or on progression/relapse, whichever is sooner, and at the time of documenting a significant (CTCAE grade > 3) IrAE.

Patients are asked to consent to allow access to any available archival tumour tissue previously obtained, as well as any excess tumour tissue acquired during the study period (as per local practice).

Patients will also be asked for permission to access any biopsies taken from planned surgery as part of their treatment, including an organ(s) affected by toxicity (e.g. liver or skin biopsy) during the course of this study, to be used for this research.

Registered household controls will be invited to attend a convenient clinic visit with the patient to provide relevant information, have a blood sample taken and receive a stool and oral swab sample collection kit.

### Sample size

No established power and sample size calculations exist for studies where the final analyses are of the nature necessitated in this project. We follow the broad approach of simulation-based power calculations. In particular, we used data based on the microbiome samples taken from the patients of the pilot study to populate the corresponding probabilities of a Dirichlet-multinomial model. These probabilities were then used to simulate 1000 cohorts of 10,000 responders (individuals with progression free survival) and 10,000 non-responders, i.e. those with progression or death. We then used the proportions of being alive and progression free or not for each of the first six cohorts and calculated how well the model would estimate the corresponding probabilities in order to estimate the power for a range of sample sizes. Table 3 gives the estimated power for three indicative cohorts and for different sample sizes. These are looking at groups of advanced melanoma patients treated with nivolumab and nivolumab+ipilimumab, where the probability of PFS after 12 months was estimated at 42 and 50% for the two cohorts respectively. The third is based on a cohort of advanced NSCLC patients treated with pembrolizumab with a 47% probability of PFS after 12 months [30].

**Table 3:** Estimated power based on cohort and sample size

	100	150	200	300	400	600
Melanoma Nivo 1 year PFS	0.650	0.682	0.710	0.738	0.754	0.759
Melanoma Nivo+IPI 1 year PFS	0.754	0.766	0.775	0.784	0.788	0.795
NSCLC Pembrolizumab 1 year PFS	0.732	0.750	0.764	0.776	0.782	0.790

It is apparent that, as expected, the power increases with the sample size, but at a slower rate. A number of approximations are used behind these calculations, with the main one relating to the lack of detailed data on the gut microbiome signature for the melanoma patients and a complete lack of data for lung and renal cancer patients. Therefore, we propose to use a minimum of 50 patients for each of the first six cohorts and re-evaluate the sample size calculations once more data become available on the gut microbiome profile. This calculation will be done when at least 50 patients from each cohort have given samples and taxonomic metagenomics and culturing methods can be applied to update the current evidence on the ability of the microbiome signature to detect the probability of responding to treatment or not.

### Statistical analysis methods

The main clinical outcome to be correlated with the gut microbiome metagenomic data will be PFS in patients with advanced disease. The hypothesis to be tested is concerned with the effect of the microbiome profile on PFS. There will be an interim analysis evaluating the effect of the biomarkers on 6-month PFS while the main analysis will assess the effect on 1 year PFS.

All primary analyses will be based upon a regularised logistic regression with the false discovery rate being controlled at 0.05 and the regularisation parameter being estimated using cross-validation.

Two sensitivity/exploratory analyses will be conducted to assess the robustness of the composition of the microbiome signature and its exact effect on the clinical outcome. The first will estimate the effect of the biomarkers on the complete survival curve (up to the particular follow-up) using a Cox model. The second will use a random forest classifier to evaluate the effect of the microbiome profile on the PFS.

Regularised logistic regression will be also used for associating the gut microbiome with a number of secondary endpoints, such as the incidence of adverse events. The secondary outcomes which depend upon the complete survival curve such the median PFS and the median OS will be correlated to the microbiome signature using a Cox model.

The logistic regression and Cox model-based analyses are likely to be conducted in R using the glmnet package. The random forest classifier will likely be fitted using the random Forest R package. A detailed statistical analysis plan will be produced before the final data base lock or before any interim analysis is performed (as appropriate).

### Interim analysis

In the first stage of this study, a total of 50 patients will be recruited in each of the first 6 cohorts with specific types of advanced cancer: melanoma, renal cancer and NSCLC and followed for a minimum of 6 months. The outcomes of this first stage will determine on-going enrolment and any requirement to amend the protocol.

### Ethics and dissemination

#### Research ethic approval

The study will be performed in accordance with the spirit and the letter of the declaration of Helsinki, the conditions and principles of Good Clinical Practice, the protocol and applicable local and national regulatory requirements and laws.

#### Dissemination of results

Ownership of the data arising from this study resides with the study management group (SMG). The main study results will be presented at national and international conferences and published in a peer-reviewed journal, on behalf of all collaborators. All participating sites and Investigators will be acknowledged in publications and presentations. In addition, patients and participants who have consented to receive updates on study progress and results, will be provided with appropriate updates and a summary of the results in lay terms.

## Discussion

ICIs are revolutionising cancer management, with growing evidence of activity across a wide spectrum of cancers. However, generally the minority of patients treated with these high-cost drugs benefit, while IrAEs can be devastating. There is an urgent need to identify biomarkers that are predictive of treatment response, resistance and toxicity, and emerging data points to the gut microbiome as an important influencer of these outcomes [7, 11].

So far, identification of specific responsible bacterial taxa has been inconsistent between published studies, which likely reflects small patient cohorts studied, as well as limitations in analytical methodologies used. By culturing and metagenomic sequencing stool sample bacteria, our group has identified a unique consortium of bacteria, which appears to be predictive of response to ICIs across all key published series as well as our own melanoma patient series [27].

MITRE is a large-scale, multicentre study on the NIHR portfolio, which will generate the largest cancer microbiome dataset of its kind. The main objective of the MITRE study is to prospectively evaluate and validate a gut microbiome ‘signature’ which can predict ICI efficacy in patients with advanced cancer, as well as its role for predicting severe ICI-induced toxicity. Collection of associated blood and tissue samples will provide a rich resource with which to interrogate immune and genomic factors that may explain the functional interplay of the microbiome and our immune system.

A number of clinical studies have demonstrated that therapeutic microbiome manipulations can be used to treat infections and autoimmune diseases; and going forward this approach may be feasible for cancer patients receiving immunotherapy. Indeed recently, as proof of principle, transplant of faecal microbiota was recently shown to promote response in a small number of ICI- refractory melanoma patients [23]. Outcomes from the MITRE study can be expected to inform future strategies for manipulating the patient microbiome with a view to enhancing treatment efficacy in the future.

## Supplementary Information

**Additional file 1.** Schedule of Assessments for cancer patients.

**Additional file 2.** Schedule of Assessments for household controls.

**Additional file 3.** Data collection details.

## Data Availability

Data sharing is not applicable to this article as no datasets were generated or analysed during the current study.

## Abbreviations

AJCC: American Joint Committee on Cancer
ALK: Anaplastic Lymphoma Kinase
ALP: Alkaline Phosphatase
BCG: Bacillus Calmette–Guérin
BMI: Body Mass Index
CCTU-CT: Cambridge Clinical Trials Unit – Cancer Theme
CD4: Cluster of Differentiation 4
CD8: Cluster of Differentiation 8
CFS: Clinical Frailty Scale
CRF: Case Report Form
CRUK: Cancer Research UK
CT: Computerized Tomography
CTCAE: Common Terminology Criteria for Adverse Events
CTLA-4: Cytotoxic T-lymphocyte-associated Protein 4
DNA: Deoxyribonucleic Acid
ECOG PS: Eastern Cooperative Oncology Group Performance Status
EGFR: Epidermal Growth Factor Receptor
CRP: C-reactive Protein
FOXP3: Forkhead Box P3
GGT: Gamma Glutamyl Transferase
GP: General Practitioner
Hb: Haemoglobin
HbA1c: Glycated Haemoglobin
IFNγ: Interferon Gamma
IL-6: Interleukin 6
IrAE: Immune-related Adverse Event
K: Potassium
KPS: Karnofsky Performance Status
LDH: Lactate Dehydrogenase
MRI: Magnetic Resonance Imaging
Na: Sodium
NCI: National Cancer Institute
NIHR: National Institute for Health Research
NSAID: Non-steroidal Anti-inflammatory Drug
NSCLC: Non-small Cell Lung Cancer
OS: Overall Survival
PBMC: Peripheral Blood Mononuclear Cell
PD-1: Programmed Cell Death Protein 1
PD-L1: Programmed Death Ligand 1
PD-(L)1: Programmed Cell Death Protein 1 or Programmed Death Ligand 1
PFS: Progression-free Survival
PPI: Proton Pump Inhibitor
REC: Research Ethics Committee
RECIST: Response Evaluation Criteria In Solid Tumours
RFS: Relapse-free Survival
RNA: Ribonucleic Acid
SARS-CoV-2: Severe Acute Respiratory Syndrome Coronavirus 2
SMG: Study Management Group
SNV: Single Nucleotide Variant
TNFα: Tumour Necrosis Factor Alpha
