# Supplementary material for: The MITRE trial protocol: a study to evaluate the microbiome as a biomarker of efficacy and toxicity in cancer patients receiving immune checkpoint inhibitor therapy
Source: BMC Cancer. 2022 Jan 24;22:99. doi: 10.1186/s12885-021-09156-x (PMC8785032; doi:10.1186/s12885-021-09156-x)
Supplement: Supplementary file 1 — Additional file 1. Schedule of Assessments for cancer patients. [file 12885_2021_9156_MOESM1_ESM.docx]

# Supplementary Information

## Appendix 1: Schedule of Assessments for cancer patients

Patients with advanced disease:

|  | **Eligible to Register** | **Baseline, pre-treatment assessments**  **(prior to first drug administration)*** | **Approx. 6-8 weeks** | **Approx. 12 weeks** | **Approx. 6 month** | **Approx. 9 month** | **Approx. 1 year** | **Approx. 6 monthly f/ups** | **Disease progression **** | **Grade >3 IrAE^~^** |
| --- | --- | --- | --- | --- | --- | --- | --- | --- | --- | --- |
| **Entry criteria met** | X |  |  |  |  |  |  |  |  |  |
| **Informed consent obtained** | X |  |  |  |  |  |  |  |  |  |
| **Clinical data collection** |  | X | X | X | X | X | X | X | X | X |
| **Routine blood samples** |  | X* | X | X | X | X | X |  | X | X |
| **Routine imaging ¤** | X* |  |  | X | X | X | X |  | X |  |
| **Stool sample¤** |  | X* | X*** | X*** | X |  | X^¥^ |  | X^¥^ | X |
| **Oral swab sample** |  | X* |  |  |  |  |  |  |  |  |
| **Research blood samples¤** |  | X* |  | X |  |  | X^¥^ |  | X^¥^ | X |
| **Nasopharyngeal swab** |  | X^§^ |  |  |  |  |  |  |  |  |
| **Tumour sample^¤** |  | X |  |  |  |  |  |  | X |  |
| **Organ biopsy^^¤** |  |  |  |  |  |  |  |  |  | X |

*Baseline routine and research blood samples, stool and oral swab samples to be collected following consent. Imaging to be performed within 45 days prior to first drug administration.

**Assessments to be captured at the point of progression. This may occur at a predefined visit in the schedule of assessments (e.g. 1 year follow-up) or at any point during the course of the study.

***Stool samples can be collected between 3-6 weeks and 9-12 weeks

^¥^ A final sample will be collected when there is either evidence of disease progression (if receiving palliative therapy), or disease relapse (if receiving adjuvant therapy), or at 1 year, whichever occurs soonest.

^~^IrAE data and samples may be collected at any time during the course of the study, whether patients are actively receiving drug or not. Results of any routine blood sample and imaging undertaken within 2 weeks of documenting a grade >3 IrAE will be requested.

^Surplus archival pre-treatment tumour tissue will be collected from all patients; any surplus tumour tissue from biopsies/surgeries undertaken during the study period will also be collected.

^^Surplus tissue collected from any organs biopsied for IrAE investigation will be collected.

¤ When the patient’s planned immunotherapy ends: If further treatment is planned with another anticancer treatment, then only data on overall survival as well as the resolution of ongoing IrAEs will be collected. No further research samples or measurements will be collected from these participants.

§ Patients who have not had any routine COVID-19 testing within 4 weeks of study entry may be offered an optional nasopharyngeal swab test for COVID-19 antigen prior to their treatment starting,

Patients receiving adjuvant therapy:

|  | **Eligible to Register** | **Baseline, pre-treatment assessments**  **(prior to first drug administration)*** | **Approx. 6-8 weeks** | **Approx.12 weeks** | **Approx.6 months** | **Approx. 9 months** | **Approx.1 year f/up**** | **Approx. 6 monthly f/up** | **Disease relapse **** | **Grade >3 IrAE^~^** |
| --- | --- | --- | --- | --- | --- | --- | --- | --- | --- | --- |
| **Entry criteria met** | X |  |  |  |  |  |  |  |  |  |
| **Informed consent** | X |  |  |  |  |  |  |  |  |  |
| **Clinical data collection** |  | X | X | X | X | X | X | X | X | X |
| **Routine blood samples** |  | X* | X | X | X | X | X |  | X | X |
| **Routine imaging¤** | X* |  |  |  | X |  | X |  | X |  |
| **Stool sample¤** |  | X* | X*** | X*** | X |  | X^¥^ |  | X^¥^ | X |
| **Oral swab sample** |  | X* |  |  |  |  |  |  |  |  |
| **Research blood samples¤** |  | X* |  | X |  |  | X^¥^ |  | X^¥^ | X |
| **Nasopharyngeal swab** |  | X^§^ |  |  |  |  |  |  |  |  |
| **Tumour sample^¤** |  | X |  |  |  |  |  |  | X |  |
| **Organ biopsy^^¤** |  |  |  |  |  |  |  |  |  | X |

*Baseline routine and research blood samples, stool and oral swab samples to be collected following consent. In the adjuvant setting, imaging to be performed within 12 weeks prior to first drug administration..

**Assessments to be captured at the point of progression. This may occur at a predefined visit in the schedule of assessments (e.g. 1 year follow-up) or at any point during the course of the study.

***Stool samples can be collected between 3-6 weeks and 9-12 weeks

^¥^ A final sample will be collected when there is either evidence of disease progression (if receiving palliative therapy), or disease relapse (if receiving adjuvant therapy), or at 1 year, whichever occurs soonest.

^~^IrAE data and samples may be collected at any time during the course of the study, whether patients are actively receiving drug or not. Results of any routine blood sample and imaging undertaken within 2 weeks of documenting a grade >3 IrAE will be requested.

^Surplus archival pre-treatment tumour tissue will be collected from all patients; any surplus tumour tissue from biopsies/surgeries undertaken during the study period will also be collected.

^^Surplus tissue collected from any organs biopsied for IrAE investigation will be collected.

¤ When the patient’s planned immunotherapy ends: If further treatment is planned with another anticancer treatment, then only data on overall survival as well as the resolution of ongoing IrAEs will be collected. No further research samples or measurements will be collected from these participants.

§ Patients who have not had any routine COVID-19 testing within 4 weeks of study entry may be offered an optional nasopharyngeal swab test for COVID-19 antigen prior to their treatment starting,
