# Supplementary material for: The MITRE trial protocol: a study to evaluate the microbiome as a biomarker of efficacy and toxicity in cancer patients receiving immune checkpoint inhibitor therapy
Source: BMC Cancer. 2022 Jan 24;22:99. doi: 10.1186/s12885-021-09156-x (PMC8785032; doi:10.1186/s12885-021-09156-x)
Supplement: Supplementary file 2 — Additional file 2. Schedule of Assessments for household controls. [file 12885_2021_9156_MOESM2_ESM.docx]

## Appendix 2: Schedule of Assessments for household controls

|  | Baseline* |
| --- | --- |
| Entry criteria | X |
| Informed consent | X |
| Clinical data collection | X |
| Stool sample | X |
| Oral swab sample | X |
| Research blood samples | X |
| Nasopharyngeal swab | X** |

*Baseline information and samples to be taken within 4 weeks of consenting

## ** Optional nasopharyngeal swab test for COVID-19 antigen may be offered to participants who have not had any routine COVID-19 testing within 4 weeks of study entry. This will be collected at the same time as blood sampling.
