# Supplementary material for: The MITRE trial protocol: a study to evaluate the microbiome as a biomarker of efficacy and toxicity in cancer patients receiving immune checkpoint inhibitor therapy
Source: BMC Cancer. 2022 Jan 24;22:99. doi: 10.1186/s12885-021-09156-x (PMC8785032; doi:10.1186/s12885-021-09156-x)
Supplement: Supplementary file 3 — Additional file 3. Data collection details. [file 12885_2021_9156_MOESM3_ESM.docx]

## Appendix 3: Data collection

## Baseline data

Information about the patient’s cancer history will be collected. This will include:

- Cancer diagnosis and staging; any associated molecular analyses (e.g. BRAF/NRAS/KRAS/EGFR/ALK/ROS1/PD-L1 status) relevant for that cancer type performed as standard of care.
- First date of diagnosis of their cancer and disease stage at first presentation, if different from that at study entry
- Any previous treatment for the patient’s cancer
- Medical and social history, including previous and current clinically important diseases including the patient’s COVID-19 status (history of any prior testing, diagnosis and treatment)
- Use of medications, including antibiotics, steroids, PPIs, NSAIDs and probiotics in the last 6 months, dietary preference (meat-eating, pescatarian, vegetarian, vegan, other)
- Demographic data, including height, weight, BMI and record approximate weight loss in the last 3 months
- AEs of special interest: anorexia, muscle weakness
- Smoking and alcohol history, family history (particularly relating to any autoimmune diseases)
- Assessment of ECOG performance status (PS), Karnofsky performance status (KPS) and Rockwood Clinical Frailty Scale (CFS)
- The most recent radiology and relevant histopathology (or cytology) reports confirming the diagnosis will be anonymised and collected.
- The most recent routine blood biochemistry and haematology test results will be anonymised and collected.
- The following list of biochemistry tests are recommended, **but NOT mandatory**, as these tests may be subject to local practice variation:
- urea, sodium (Na), potassium (K), gamma glutamyl transferase (GGT), serum ALT or serum AST, total bilirubin, albumin, total protein, creatinine, serum calcium, alkaline phosphatase (ALP), lactate dehydrogenase (LDH), phosphate, magnesium, thyroid function tests, HbA1C, serum glucose, random cortisol, CRP
- The following list of haematology tests **are mandatory**:
- haemoglobin (Hb), white cell count including differential, neutrophil, lymphocyte and eosinophil count, platelet count.
- Any auto-antibody tests undertaken will be recorded

## On-treatment data collection

Prospectively, at approximately 6-8 weeks, 12 weeks, 6 months, 9 months, 1 year, then 6 monthly thereafter until disease progression or relapse, the following clinical data will be collected, to include:

- Haematology and Biochemistry tests as per local practice
- Any imaging undertaken (modality, extent of imaging and dates)
- RECIST tumour measurements (only at designated scanning time points)
- Any changes in anticancer treatment
- IrAEs (See Section 12.2.1)
- Antibiotic, steroid, PPI, NSAID and probiotic use
- Weight
- AEs of special interest: anorexia, muscle weakness
- Performance status (ECOG PS, KPS, Rockwood CFS)
- Any changes regarding the patient’s COVID-19 status (including new testing, diagnosis and treatment)
- Development of any new medical conditions, in particular any which may be related to autoimmunity

## Off-treatment data

At disease progression or relapse, sites of disease spread and subsequent planned anticancer treatment will be documented. Patients will then be followed only for resolution of any ongoing IrAEs of CTCAE grade ≥2, and for overall survival. New IrAEs during the course of the study will only be collected if the patient does not receive any further systemic anticancer therapy.

If treatment is stopped or delayed for any reason other than progression or relapse then on-treatment data collection should be adopted until disease progression or relapse. If subsequent systemic anticancer therapy is planned, this will be documented and patients will then be followed only for resolution of any ongoing IrAEs of CTCAE grade ≥2 and for overall survival.
